# [(*E*)-10-(2,6-Dimethyl­phenyl­imino)-9-methyl-9,10-dihydro­phenanthren-9-olato]penta­methyl­dialuminum(III)

**DOI:** 10.1107/S1600536811036312

**Published:** 2011-09-14

**Authors:** Bo Gao, Qing Su, Wei Gao, Ying Mu

**Affiliations:** a State Key Laboratory of Supramolecular Structure and Materials, School of Chemistry, Jilin University, Changchun 130012, People’s Republic of China; b School of Chemistry, Jilin University, Changchun 130012, People’s Republic of China

## Abstract

The two Al atoms in the title compound, [Al_2_(CH_3_)_5_(C_23_H_20_NO)], are four-coordinated in a distorted tetra­hedral environment. The coordination of one Al atom includes three methyl-C atoms and the O atom from the ligand, whereas the second Al atom is surrounded by the O atom and one N atom from the ligand as well as by two methyl-C atoms. In the ligand, the dihedral angle between the two phenyl rings in the 9,10-dihydro­phenanthren unit is 20.64 (12)°.

## Related literature

For background to Al complexes, see: Wang *et al.* (2006 ▶); Evans (1993 ▶); Liu *et al.* (2005 ▶, 2006 ▶); Yao *et al.* (2008 ▶); Gao *et al.* (2009 ▶). For background to anilido–imine complexes, see: Liu *et al.* (2005 ▶, 2006 ▶); Ren *et al.* (2007 ▶); Su *et al.* (2007 ▶); Yao *et al.* (2008 ▶); Wang *et al.* (2006 ▶). For the synthesis of the ligand, see: Li (2009 ▶).
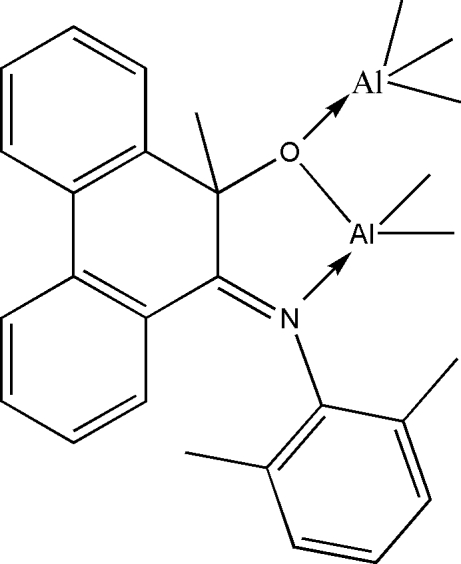


## Experimental

### 

#### Crystal data

[Al_2_(CH_3_)_5_(C_23_H_20_NO)]
*M* *_r_* = 455.53
Triclinic, 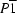

*a* = 10.4535 (17) Å
*b* = 11.4306 (18) Å
*c* = 12.221 (2) Å
α = 84.930 (3)°
β = 86.308 (3)°
γ = 64.092 (2)°
*V* = 1307.9 (4) Å^3^
*Z* = 2
Mo *K*α radiation
μ = 0.13 mm^−1^
*T* = 185 K
0.36 × 0.32 × 0.19 mm

#### Data collection

SMART CCD area-detector diffractometer
Absorption correction: multi-scan (*SADABS*; Bruker, 2001 ▶) *T*_min_ = 0.955, *T*_max_ = 0.976
6899 measured reflections
4966 independent reflections
3558 reflections with *I* > 2σ(*I*)
*R*_int_ = 0.020

#### Refinement

*R*[*F* ^2^ > 2σ(*F* ^2^)] = 0.054
*wR*(*F* ^2^) = 0.150
*S* = 1.03
4966 reflections
297 parameters
H-atom parameters constrained
Δρ_max_ = 0.48 e Å^−3^
Δρ_min_ = −0.36 e Å^−3^

### 

Data collection: *SMART* (Bruker, 1998 ▶); cell refinement: *SAINT* (Bruker, 1998 ▶); data reduction: *SAINT*; program(s) used to solve structure: *SHELXS97* (Sheldrick, 2008 ▶); program(s) used to refine structure: *SHELXL97* (Sheldrick, 2008 ▶); molecular graphics: *XP* in *SHELXTL* (Sheldrick, 2008 ▶); software used to prepare material for publication: *SHELXTL*.

## Supplementary Material

Crystal structure: contains datablock(s) I. DOI: 10.1107/S1600536811036312/wm2522sup1.cif

Structure factors: contains datablock(s) I. DOI: 10.1107/S1600536811036312/wm2522Isup2.hkl

Additional supplementary materials:  crystallographic information; 3D view; checkCIF report

## Figures and Tables

**Table 1:** Selected bond lengths (Å)

Al1—O1	1.9273 (17)
Al1—C25	1.972 (3)
Al1—C26	1.974 (3)
Al1—C24	1.980 (3)
Al2—O1	1.8552 (17)
Al2—C28	1.944 (3)
Al2—C27	1.954 (3)
Al2—N1	1.993 (2)

**Fig. 1.**
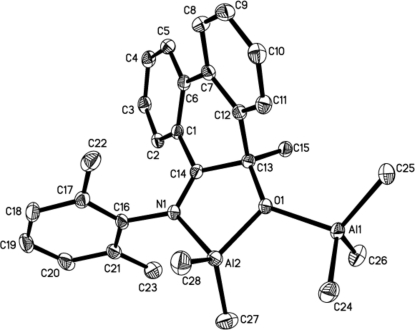
View of the molecule of compound (I) showing the atom labelling scheme, with displacement ellipsoids drawn at the 30% probability level. Hydrogen atoms were omitted for clarity.

## supplementary crystallographic information

### Comment

Organoaluminum complexes have received considerable attention due to their interesting properties and potential applications in organic synthesis and catalysis. It is known that alkylaluminum reagents are widely applied to Lewis acid-mediated reactions while aluminium acetylides play an important role in addition reaction (Evans, 1993). Organoaluminum complexes supported by anilido-imine, β-diketiminate and salicyaldiminato ligands are of particular interest, owning to their interesting coordination chemistry and catalytic performance (Wang *et al.*, 2006). Furthermore, we have previously reported a series of Zn(II) (Su *et al.*, 2007), Al(III) (Liu *et al.*, 2005; 2006; Yao *et al.*, 2008) and B(III) (Ren *et al.*, 2007) complexes with chelating anilido-imine ligands. As a part of our continuing study, we have investigated the two-step reaction procedures including the 1,2-addition reaction of trimethylaluminium with (*E*)-10-(2,6-dimethylphenylimino)phenanthren-9(10*H*)-one), and subseqent reaction with trimethylaluminium to form the corresponding product. Herein, the preparation and crystal structure of the title compound, (I), [Al_2_(CH_3_)_5_(C_23_H_20_NO)], is reported.

In the molecule of compound (I), (Fig. 1), the two Al atoms exist in different coordination environments, both adopting distorted tetrahedral geometries. The tetrahedral coordination around Al1 involves three methyl-C atoms and the O1 atom from the ligand. The coordination around the Al2 atom involves the O1 atom and N1 atom from the ligand and two methyl-C atoms. The Al—Al separation distance is 3.1625 (13) Å. The Al2—O1 distance (1.8552 (17) Å) is significantly shorter than the Al1—O1 distance (1.9273 (17) Å), indicating that the former has a more covalent character. The two Al2—O1 and Al1—O1 distances are somewhat longer than the corresponding distances in {µ-[2-(dimethylamino)phenyl] (2-fluorophenyl)methanolato}pentamethyldialuminum(III) (Gao *et al.*, 2009; Al2—O1, 1.8165 (19) Å; Al1—O1, 1.9199 (19) Å), owing to a larger steric disturbance. The five-membered chelate ring, O1/Al2/N1/C13/C14, is nearly planar, with a maximum deviation of 0.059 (2) Å of O1 from the least-squares plane. The dihedral angles between the five-membered chelate ring and the phenyl rings C16—C11, C7—C11 and C1—C6 are 82.78 (12)°, 62.74 (11) ° and 46.99 (11) °, respectively. The coplarity of the 9,10-phenanthrene aromatic rings is not retained after the addition reaction of Al(CH_3_)_3_ to the C═O bond of (*E*)-10-(2,6-dimethylphenylimino)phenanthren-9(10*H*)-one) with the dihedral angle between the two phenyl rings (C7—C12, C1—C6) being 20.64 (12)°.

### Experimental

The dinuclear aluminium complex was prepared as following. The Schiff base ligand ((*E*)-10-(2,6-dimethylphenylimino)phenanthren-9(10*H*)-one) (1.0 mmol) which was synthesized according to the reported literature (Li, 2009), was dissolved in toluene (20 ml), and then trimethylaluminum (1.1 mmol) in hexane solution (1.1 ml, 1*M*) was added slowly at 243 K. The whole mixture was warmed up to room temperature in 2 h and refluxed for 5 h which provided a clear, yellow solution. Then volatile materials were removed under vacuum. The residue was recrystallized in toluene to give yellow crystalline solid (yield: 61%, 0.277 g). Anal. Calcd. for C_28_H_35_Al_2_NO (455.55): C 73.82, H 7.74, N 3.07; Found: C 73.80, H 7.72, N 3.04%. ^1^H NMR (300 MHz, CDCl_3_, 298 K) δ (p.p.m.): -0.93 (s, 3H,Al(CH_3_)_2_), -0.65(s, 9H,Al(CH_3_)_3_), -0.45 (s, 3H,Al(CH_3_)_2_), 1.41(s, 3H, Ar(CH_3_)_2_), 1.98 (s, 3H, CH_3_), 2.44 (s, 3H, Ar (CH_3_)_2_), 6.65 (m, 1H), 6.88 (m, 1H), 7.05 (m, 1H), 7.12 (m, 1H), 7.19 (m, 1H), 7.44 (m, 2H), 7.55 (m, 1H), 7.69 (m, 1H), 7.84 (m, 1H), 7.95 (m, 1H). ^13^C NMR (75 MHz, CDCl_3_, 298 K) δ (p.p.m.): -8.8,-7.2,-5.1,16.9, 18.2, 18.8, 30.6, 48.3, 123.9, 124.3, 124.8, 126.0, 126.4, 127.3, 127.6, 128.2, 128.6, 128.7, 129.1, 129.2, 129.3, 131.3, 131.8, 132.5, 133.9, 136.6.

### Refinement

The C-bound H atoms were positioned geometrically with C—H = 0.93 (aromatic carbon) and 0.96 (methyl) Å, and allowed to ride on their parent atoms in the riding model approximation with *U*_iso_(H) = 1.2 (1.5 for methyl) *U*_eq_(C).

### Crystal data

**Table:**

[Al_2_(CH_3_)_5_(C_23_H_20_NO)]	*Z* = 2
*M**_r_* = 455.53	*F*(000) = 488
Triclinic, *P*1	*D*_x_ = 1.157 Mg m^−^^3^
Hall symbol: -P 1	Mo *K*α radiation, λ = 0.71073 Å
*a* = 10.4535 (17) Å	Cell parameters from 2148 reflections
*b* = 11.4306 (18) Å	θ = 3.6–52.0°
*c* = 12.221 (2) Å	µ = 0.13 mm^−^^1^
α = 84.930 (3)°	*T* = 185 K
β = 86.308 (3)°	Block, colorless
γ = 64.092 (2)°	0.36 × 0.32 × 0.19 mm
*V* = 1307.9 (4) Å^3^	

### Data collection

**Table:**

SMART CCD area-detector diffractometer	4966 independent reflections
Radiation source: fine-focus sealed tube	3558 reflections with *I* > 2σ(*I*)
graphite	*R*_int_ = 0.020
phi and ω scans	θ_max_ = 26.0°, θ_min_ = 1.7°
Absorption correction: multi-scan (*SADABS*; Bruker, 2001)	*h* = −11→12
*T*_min_ = 0.955, *T*_max_ = 0.976	*k* = −11→14
6899 measured reflections	*l* = −15→14

### Refinement

**Table:**

Refinement on *F*^2^	Primary atom site location: structure-invariant direct methods
Least-squares matrix: full	Secondary atom site location: difference Fourier map
*R*[*F*^2^ > 2σ(*F*^2^)] = 0.054	Hydrogen site location: inferred from neighbouring sites
*wR*(*F*^2^) = 0.150	H-atom parameters constrained
*S* = 1.03	*w* = 1/[σ^2^(*F*_o_^2^) + (0.0783*P*)^2^ + 0.4595*P*] where *P* = (*F*_o_^2^ + 2*F*_c_^2^)/3
4966 reflections	(Δ/σ)_max_ < 0.001
297 parameters	Δρ_max_ = 0.48 e Å^−^^3^
0 restraints	Δρ_min_ = −0.36 e Å^−^^3^

### Special details

**Table:**

Experimental. see experiment
Geometry. All e.s.d.'s (except the e.s.d. in the dihedral angle between two l.s. planes) are estimated using the full covariance matrix. The cell e.s.d.'s are taken into account individually in the estimation of e.s.d.'s in distances, angles and torsion angles; correlations between e.s.d.'s in cell parameters are only used when they are defined by crystal symmetry. An approximate (isotropic) treatment of cell e.s.d.'s is used for estimating e.s.d.'s involving l.s. planes.
Refinement. Refinement of *F*^2^ against ALL reflections. The weighted *R*-factor *wR* and goodness of fit *S* are based on *F*^2^, conventional *R*-factors *R* are based on *F*, with *F* set to zero for negative *F*^2^. The threshold expression of *F*^2^ > σ(*F*^2^) is used only for calculating *R*-factors(gt) *etc*. and is not relevant to the choice of reflections for refinement. *R*-factors based on *F*^2^ are statistically about twice as large as those based on *F*, and *R*- factors based on ALL data will be even larger.

### Fractional atomic coordinates and isotropic or equivalent isotropic displacement parameters (Å^2^)

**Table:**

	*x*	*y*	*z*	*U*_iso_*/*U*_eq_	
Al1	−0.25584 (8)	0.40105 (7)	0.13162 (7)	0.0300 (2)	
Al2	−0.06773 (8)	0.36784 (8)	0.33504 (6)	0.0288 (2)	
O1	−0.08466 (16)	0.29493 (15)	0.21020 (13)	0.0250 (4)	
N1	0.1395 (2)	0.25468 (19)	0.31395 (16)	0.0253 (5)	
C1	0.3077 (2)	0.0805 (2)	0.19939 (19)	0.0252 (5)	
C2	0.4290 (3)	0.1021 (3)	0.1830 (2)	0.0290 (6)	
H2	0.4254	0.1823	0.1968	0.035*	
C3	0.5548 (3)	0.0042 (3)	0.1463 (2)	0.0334 (6)	
H3	0.6355	0.0188	0.1336	0.040*	
C4	0.5597 (3)	−0.1155 (3)	0.1285 (2)	0.0353 (6)	
H4	0.6451	−0.1822	0.1060	0.042*	
C5	0.4395 (3)	−0.1376 (3)	0.1436 (2)	0.0323 (6)	
H5	0.4449	−0.2187	0.1307	0.039*	
C6	0.3106 (3)	−0.0396 (2)	0.17803 (19)	0.0269 (5)	
C7	0.1771 (3)	−0.0561 (2)	0.19599 (19)	0.0266 (5)	
C8	0.1793 (3)	−0.1788 (2)	0.2152 (2)	0.0341 (6)	
H8	0.2659	−0.2527	0.2143	0.041*	
C9	0.0543 (3)	−0.1924 (3)	0.2355 (2)	0.0360 (6)	
H9	0.0570	−0.2750	0.2461	0.043*	
C10	−0.0745 (3)	−0.0827 (3)	0.2400 (2)	0.0347 (6)	
H10	−0.1581	−0.0917	0.2555	0.042*	
C11	−0.0793 (3)	0.0401 (3)	0.2216 (2)	0.0308 (6)	
H11	−0.1663	0.1133	0.2251	0.037*	
C12	0.0445 (3)	0.0554 (2)	0.19813 (19)	0.0252 (5)	
C13	0.0425 (2)	0.1901 (2)	0.17092 (19)	0.0235 (5)	
C14	0.1680 (2)	0.1824 (2)	0.23304 (19)	0.0238 (5)	
C15	0.0633 (3)	0.2121 (2)	0.0467 (2)	0.0290 (6)	
H15A	−0.0174	0.2171	0.0096	0.044*	
H15B	0.1479	0.1409	0.0213	0.044*	
H15C	0.0723	0.2920	0.0315	0.044*	
C16	0.2479 (2)	0.2390 (3)	0.3914 (2)	0.0293 (6)	
C17	0.2796 (3)	0.1390 (3)	0.4746 (2)	0.0370 (7)	
C18	0.3754 (3)	0.1313 (3)	0.5532 (2)	0.0490 (9)	
H18	0.3996	0.0659	0.6096	0.059*	
C19	0.4342 (3)	0.2176 (3)	0.5490 (3)	0.0529 (9)	
H19	0.4959	0.2111	0.6032	0.064*	
C20	0.4026 (3)	0.3144 (3)	0.4649 (3)	0.0462 (8)	
H20	0.4444	0.3716	0.4623	0.055*	
C21	0.3079 (3)	0.3267 (3)	0.3836 (2)	0.0343 (6)	
C22	0.2180 (3)	0.0417 (3)	0.4797 (2)	0.0471 (8)	
H22A	0.1166	0.0859	0.4905	0.071*	
H22B	0.2580	−0.0219	0.5397	0.071*	
H22C	0.2400	−0.0009	0.4121	0.071*	
C23	0.2785 (3)	0.4301 (3)	0.2915 (3)	0.0416 (7)	
H23A	0.3604	0.4064	0.2428	0.062*	
H23B	0.2578	0.5117	0.3211	0.062*	
H23C	0.1984	0.4382	0.2518	0.062*	
C24	−0.3850 (3)	0.4894 (3)	0.2547 (3)	0.0456 (8)	
H24A	−0.3840	0.4254	0.3115	0.068*	
H24B	−0.4799	0.5379	0.2287	0.068*	
H24C	−0.3538	0.5477	0.2835	0.068*	
C25	−0.3291 (3)	0.2930 (3)	0.0638 (3)	0.0489 (8)	
H25A	−0.2523	0.2249	0.0271	0.073*	
H25B	−0.3996	0.3463	0.0115	0.073*	
H25C	−0.3711	0.2552	0.1197	0.073*	
C26	−0.2137 (3)	0.5213 (3)	0.0258 (3)	0.0478 (8)	
H26A	−0.1576	0.5546	0.0603	0.072*	
H26B	−0.3011	0.5923	0.0023	0.072*	
H26C	−0.1617	0.4754	−0.0367	0.072*	
C27	−0.0914 (3)	0.5476 (3)	0.3150 (3)	0.0488 (8)	
H27A	−0.0640	0.5645	0.2410	0.073*	
H27B	−0.0326	0.5605	0.3653	0.073*	
H27C	−0.1892	0.6061	0.3289	0.073*	
C28	−0.1394 (3)	0.3076 (3)	0.4682 (2)	0.0499 (8)	
H28A	−0.2332	0.3720	0.4864	0.075*	
H28B	−0.0774	0.2940	0.5275	0.075*	
H28C	−0.1432	0.2272	0.4566	0.075*	

### Atomic displacement parameters (Å^2^)

**Table:**

	*U*^11^	*U*^22^	*U*^33^	*U*^12^	*U*^13^	*U*^23^
Al1	0.0220 (4)	0.0301 (4)	0.0366 (4)	−0.0095 (3)	−0.0072 (3)	−0.0015 (3)
Al2	0.0225 (4)	0.0305 (4)	0.0322 (4)	−0.0090 (3)	−0.0017 (3)	−0.0079 (3)
O1	0.0195 (8)	0.0269 (9)	0.0283 (9)	−0.0090 (7)	−0.0039 (7)	−0.0031 (7)
N1	0.0210 (10)	0.0268 (11)	0.0276 (11)	−0.0090 (9)	−0.0056 (8)	−0.0026 (8)
C1	0.0198 (12)	0.0293 (13)	0.0224 (12)	−0.0065 (10)	−0.0025 (9)	−0.0018 (10)
C2	0.0259 (13)	0.0326 (14)	0.0292 (13)	−0.0125 (11)	−0.0067 (10)	−0.0027 (10)
C3	0.0203 (12)	0.0446 (16)	0.0322 (14)	−0.0109 (12)	−0.0025 (10)	−0.0032 (12)
C4	0.0218 (13)	0.0404 (16)	0.0334 (14)	−0.0032 (12)	−0.0017 (11)	−0.0059 (12)
C5	0.0293 (14)	0.0303 (14)	0.0341 (14)	−0.0091 (11)	−0.0039 (11)	−0.0044 (11)
C6	0.0250 (13)	0.0292 (13)	0.0236 (12)	−0.0090 (11)	−0.0043 (10)	0.0000 (10)
C7	0.0283 (13)	0.0297 (14)	0.0221 (12)	−0.0126 (11)	−0.0018 (10)	−0.0030 (10)
C8	0.0346 (15)	0.0252 (14)	0.0379 (15)	−0.0086 (12)	−0.0028 (12)	−0.0016 (11)
C9	0.0450 (17)	0.0295 (15)	0.0391 (16)	−0.0216 (13)	−0.0042 (13)	0.0010 (11)
C10	0.0357 (15)	0.0413 (16)	0.0344 (15)	−0.0243 (13)	−0.0035 (12)	0.0034 (12)
C11	0.0264 (13)	0.0343 (15)	0.0323 (14)	−0.0139 (11)	−0.0028 (11)	0.0007 (11)
C12	0.0267 (13)	0.0262 (13)	0.0238 (12)	−0.0124 (11)	−0.0028 (10)	−0.0013 (10)
C13	0.0182 (11)	0.0239 (12)	0.0274 (13)	−0.0078 (10)	−0.0012 (9)	−0.0028 (9)
C14	0.0227 (12)	0.0235 (12)	0.0261 (12)	−0.0113 (10)	−0.0041 (10)	0.0028 (9)
C15	0.0258 (13)	0.0310 (14)	0.0286 (13)	−0.0106 (11)	−0.0032 (10)	−0.0014 (10)
C16	0.0192 (12)	0.0353 (15)	0.0275 (13)	−0.0045 (11)	−0.0044 (10)	−0.0098 (11)
C17	0.0312 (14)	0.0365 (15)	0.0284 (14)	−0.0001 (12)	−0.0027 (11)	−0.0055 (11)
C18	0.0433 (17)	0.0500 (19)	0.0288 (15)	0.0046 (15)	−0.0106 (13)	−0.0048 (13)
C19	0.0350 (16)	0.064 (2)	0.0409 (18)	0.0012 (16)	−0.0165 (13)	−0.0203 (16)
C20	0.0278 (15)	0.057 (2)	0.0526 (19)	−0.0130 (14)	−0.0085 (13)	−0.0239 (16)
C21	0.0234 (13)	0.0384 (16)	0.0377 (15)	−0.0077 (12)	−0.0043 (11)	−0.0130 (12)
C22	0.0500 (18)	0.0384 (17)	0.0394 (17)	−0.0088 (15)	0.0020 (14)	0.0058 (13)
C23	0.0346 (16)	0.0386 (17)	0.0578 (19)	−0.0208 (13)	−0.0055 (14)	−0.0047 (14)
C24	0.0261 (14)	0.0463 (18)	0.059 (2)	−0.0099 (13)	−0.0006 (13)	−0.0086 (15)
C25	0.0359 (16)	0.0481 (18)	0.062 (2)	−0.0137 (14)	−0.0192 (15)	−0.0092 (15)
C26	0.0395 (17)	0.0454 (18)	0.0518 (19)	−0.0136 (14)	−0.0099 (14)	0.0106 (14)
C27	0.0315 (15)	0.0367 (17)	0.080 (2)	−0.0140 (13)	−0.0009 (15)	−0.0177 (16)
C28	0.0411 (17)	0.063 (2)	0.0366 (16)	−0.0146 (16)	0.0030 (13)	−0.0062 (14)

### Geometric parameters (Å, °)

**Table:**

Al1—O1	1.9273 (17)	C15—H15A	0.9600
Al1—C25	1.972 (3)	C15—H15B	0.9600
Al1—C26	1.974 (3)	C15—H15C	0.9600
Al1—C24	1.980 (3)	C16—C21	1.389 (4)
Al2—O1	1.8552 (17)	C16—C17	1.399 (4)
Al2—C28	1.944 (3)	C17—C18	1.401 (4)
Al2—C27	1.954 (3)	C17—C22	1.504 (4)
Al2—N1	1.993 (2)	C18—C19	1.367 (5)
O1—C13	1.436 (3)	C18—H18	0.9300
N1—C14	1.281 (3)	C19—C20	1.383 (5)
N1—C16	1.465 (3)	C19—H19	0.9300
C1—C2	1.393 (3)	C20—C21	1.403 (4)
C1—C6	1.407 (3)	C20—H20	0.9300
C1—C14	1.477 (3)	C21—C23	1.499 (4)
C2—C3	1.382 (3)	C22—H22A	0.9600
C2—H2	0.9300	C22—H22B	0.9600
C3—C4	1.382 (4)	C22—H22C	0.9600
C3—H3	0.9300	C23—H23A	0.9600
C4—C5	1.384 (4)	C23—H23B	0.9600
C4—H4	0.9300	C23—H23C	0.9600
C5—C6	1.392 (3)	C24—H24A	0.9600
C5—H5	0.9300	C24—H24B	0.9600
C6—C7	1.489 (3)	C24—H24C	0.9600
C7—C8	1.391 (4)	C25—H25A	0.9600
C7—C12	1.415 (3)	C25—H25B	0.9600
C8—C9	1.386 (4)	C25—H25C	0.9600
C8—H8	0.9300	C26—H26A	0.9600
C9—C10	1.384 (4)	C26—H26B	0.9600
C9—H9	0.9300	C26—H26C	0.9600
C10—C11	1.381 (4)	C27—H27A	0.9600
C10—H10	0.9300	C27—H27B	0.9600
C11—C12	1.388 (3)	C27—H27C	0.9600
C11—H11	0.9300	C28—H28A	0.9600
C12—C13	1.538 (3)	C28—H28B	0.9600
C13—C14	1.523 (3)	C28—H28C	0.9600
C13—C15	1.534 (3)		
			
O1—Al1—C25	111.41 (11)	H15A—C15—H15B	109.5
O1—Al1—C26	107.52 (11)	C13—C15—H15C	109.5
C25—Al1—C26	113.94 (15)	H15A—C15—H15C	109.5
O1—Al1—C24	100.23 (11)	H15B—C15—H15C	109.5
C25—Al1—C24	109.59 (14)	C21—C16—C17	123.2 (2)
C26—Al1—C24	113.30 (14)	C21—C16—N1	119.6 (2)
O1—Al2—C28	113.23 (12)	C17—C16—N1	117.1 (2)
O1—Al2—C27	116.12 (12)	C16—C17—C18	116.5 (3)
C28—Al2—C27	120.52 (15)	C16—C17—C22	122.6 (2)
O1—Al2—N1	84.06 (8)	C18—C17—C22	120.8 (3)
C28—Al2—N1	109.79 (12)	C19—C18—C17	121.7 (3)
C27—Al2—N1	106.33 (11)	C19—C18—H18	119.2
C13—O1—Al2	116.20 (13)	C17—C18—H18	119.2
C13—O1—Al1	128.09 (14)	C18—C19—C20	120.5 (3)
Al2—O1—Al1	113.45 (9)	C18—C19—H19	119.7
C14—N1—C16	121.9 (2)	C20—C19—H19	119.7
C14—N1—Al2	113.25 (16)	C19—C20—C21	120.4 (3)
C16—N1—Al2	124.23 (15)	C19—C20—H20	119.8
C2—C1—C6	121.0 (2)	C21—C20—H20	119.8
C2—C1—C14	122.9 (2)	C16—C21—C20	117.6 (3)
C6—C1—C14	116.0 (2)	C16—C21—C23	123.0 (2)
C3—C2—C1	119.9 (2)	C20—C21—C23	119.4 (3)
C3—C2—H2	120.1	C17—C22—H22A	109.5
C1—C2—H2	120.1	C17—C22—H22B	109.5
C2—C3—C4	119.5 (2)	H22A—C22—H22B	109.5
C2—C3—H3	120.2	C17—C22—H22C	109.5
C4—C3—H3	120.2	H22A—C22—H22C	109.5
C3—C4—C5	121.1 (2)	H22B—C22—H22C	109.5
C3—C4—H4	119.5	C21—C23—H23A	109.5
C5—C4—H4	119.5	C21—C23—H23B	109.5
C4—C5—C6	120.5 (2)	H23A—C23—H23B	109.5
C4—C5—H5	119.7	C21—C23—H23C	109.5
C6—C5—H5	119.7	H23A—C23—H23C	109.5
C5—C6—C1	118.0 (2)	H23B—C23—H23C	109.5
C5—C6—C7	123.6 (2)	Al1—C24—H24A	109.5
C1—C6—C7	118.4 (2)	Al1—C24—H24B	109.5
C8—C7—C12	118.8 (2)	H24A—C24—H24B	109.5
C8—C7—C6	121.6 (2)	Al1—C24—H24C	109.5
C12—C7—C6	119.6 (2)	H24A—C24—H24C	109.5
C9—C8—C7	120.9 (2)	H24B—C24—H24C	109.5
C9—C8—H8	119.5	Al1—C25—H25A	109.5
C7—C8—H8	119.5	Al1—C25—H25B	109.5
C10—C9—C8	119.8 (2)	H25A—C25—H25B	109.5
C10—C9—H9	120.1	Al1—C25—H25C	109.5
C8—C9—H9	120.1	H25A—C25—H25C	109.5
C11—C10—C9	120.2 (3)	H25B—C25—H25C	109.5
C11—C10—H10	119.9	Al1—C26—H26A	109.5
C9—C10—H10	119.9	Al1—C26—H26B	109.5
C10—C11—C12	120.7 (2)	H26A—C26—H26B	109.5
C10—C11—H11	119.7	Al1—C26—H26C	109.5
C12—C11—H11	119.7	H26A—C26—H26C	109.5
C11—C12—C7	119.5 (2)	H26B—C26—H26C	109.5
C11—C12—C13	122.1 (2)	Al2—C27—H27A	109.5
C7—C12—C13	118.3 (2)	Al2—C27—H27B	109.5
O1—C13—C14	108.52 (18)	H27A—C27—H27B	109.5
O1—C13—C15	110.23 (19)	Al2—C27—H27C	109.5
C14—C13—C15	111.17 (19)	H27A—C27—H27C	109.5
O1—C13—C12	113.07 (18)	H27B—C27—H27C	109.5
C14—C13—C12	103.48 (19)	Al2—C28—H28A	109.5
C15—C13—C12	110.20 (19)	Al2—C28—H28B	109.5
N1—C14—C1	127.7 (2)	H28A—C28—H28B	109.5
N1—C14—C13	117.1 (2)	Al2—C28—H28C	109.5
C1—C14—C13	114.8 (2)	H28A—C28—H28C	109.5
C13—C15—H15A	109.5	H28B—C28—H28C	109.5
C13—C15—H15B	109.5		
